# Overtransfusion of Autologous Blood Identifies Opportunities for Improving Patient Blood Management

**DOI:** 10.7759/cureus.4202

**Published:** 2019-03-07

**Authors:** Alejandro Perez, Sara Bakhtary, Elena Nedelcu, Solmaz Manuel

**Affiliations:** 1 Miscellaneous, University of California, San Francisco, USA; 2 Pathology, University of California, San Francisco, USA; 3 Anesthesiology, University of California, San Francisco, USA

**Keywords:** transfusion, blood management, autologous blood, preoperative autologous blood donation, wastage, anemia

## Abstract

Background

Preoperative autologous blood donation (PABD) has been declining in use nationally. A subset of patients scheduled for elective surgery, however, continue to be offered and choose this option. Our study aimed to understand the current impact of PABD before scheduled surgical procedures.

Study design and methods

A retrospective review was conducted in a single large academic center. Medical records associated with autologous units received in the transfusion service over a two-year period (1/1/2016-12/31/2017) were reviewed. Demographics, units donated, units transfused, wastage, pre-donation hemoglobin (Hb), pre-operative Hb, estimated blood loss (EBL), and clinical specialty were collected.

Results

During the study period, 118 patients underwent PABD, donating a total of 141 autologous red blood cell units. Patients who donated autologous units and were subsequently transfused had lower pre-donation Hb compared to patients who were not transfused (13.3 ± 1.4 g/dL vs. 14.3 ± 1.5 g/dL, p=0.004). Pre-operative Hb was lower than pre-donation Hb among both groups (12.1 ± 1.2 g/dL for patients receiving transfusion; 12.9 ± 1.5 g/dL, p=0.011 for patients who were not transfused). The majority of PABD patients (71%) had an estimated blood loss of less than 500 mL. Wastage rate of autologous units was 67%. PABD was disproportionately associated with a minority of surgeons and clinical services.

Conclusion

Within our institution, PABD is heavily used amongst a small subset of physicians across multiple surgical specialties and is associated with lower pre-operative Hb, tendency towards overtransfusion, and high rates of wastage of donated units. Our findings reinforce reports of inefficiencies in patient blood management and increased risks to patient health associated with PABD.

## Introduction

Preoperative autologous blood donation (PABD) involves patients donating one or more units of their own blood in the weeks preceding scheduled operative procedures. Donated units are stored and transfused to the donor as needed during or after the scheduled procedure. PABD gained significant traction during the 1980s and 1990s after it was recognized that HIV and hepatitis C virus (HCV) could be transmitted by allogeneic blood transfusion [1]. Since that time, however, widespread public measures including rigorous pre-donor screening and increased donor blood testing have significantly reduced the risk of transfusion-transmitted HIV or hepatitis infection. In the United States, the current risk of transmission per unit blood of HIV is approximately 1 in 1.5 to 2 million and for HCV 1 in 1 to 2 million [2].

At the same time, concerns have arisen regarding the safety of PABD after multiple studies have reported that autologous blood donors present with reduced preoperative hemoglobin (Hb) [3-6] and are at increased risk of perioperative blood transfusion. Preoperative anemia has also been associated with increased 30-day morbidity and mortality [7-9] in both cardiac and non-cardiac patients.

Recent recommendations also encourage restrictive transfusion practices in recognition of the lack of benefit of more liberal transfusion in most patient populations [10]. In recognition of accumulating evidence supporting restrictive transfusion practices, the already high fraction of discarded autologous blood is only expected to increase in the future [11].

Despite these concerns and projections, PABD remains a common practice at some hospitals. In this study, we sought to better understand the impact of PABD on our institution’s participating patients.

## Materials and methods

After receiving Institutional Review Board approval, we retrospectively analyzed the medical records of individuals who donated one or more autologous blood units prior to scheduled surgical procedures at a single large academic medical center between January 1, 2016 and December 31, 2017. 

The following data were collected from donors’ medical records: demographics, surgical service, and surgery type; estimated blood loss (EBL); Hb values, and transfusion of any blood products. Descriptive and univariate statistical analyses were performed using STATA 15.1 (StataCorp LP Texas, USA). Continuous variables are expressed as means with standard deviations (SD). T-tests were used for comparison between groups after assurance of normal distribution. Categorical data are expressed as ratios and compared using the Fisher’s exact test. P<0.05 was considered statistically significant.

## Results

During the study period, 118 patients underwent PABD donating a total of 141 autologous RBC units. Of these 118 patients, 83% donated a single unit with the other 17% donating between 2-5 units (19 donated 2 units, 1 donated 5 units). Surgery was canceled or postponed for 9% (11) of PABD patients for reasons unrelated to blood donation or anemia. For those that underwent surgery, the mean time from autologous blood donation to surgical procedure was 18.8 days (range 3 to 58 days). Characteristics of donors are described in Table 1.

**Table 1:** Characteristics and Hb Values of Autologous Blood Donors who were Transfused versus Not Transfused Abbreviations: Hb, hemoglobin; SD, standard deviation * Denotes significance; p-value < 0.05

	All (n=118)	Not Transfused (n=70)	Transfused (n=41)	P
Autologous blood transfusion		70 (63.1%)	41 (36.9%)	
Age, years	45.3 (±16.1)	47.3 (±15.6)	43.3 (±17.2)	0.211
Gender (female/male)	60/58	32/38	23/18	0.291
Estimated blood loss, mean (SD), mL	350.8 (± 350.9)	239.5 (±196.8)	550.8(±464.4)	0.001*
Allogeneic blood transfusion	6 (5.4%)	0 (0%)	6 (14.6%)	0.001*
Predonation Hb, mean (SD), g/dL	13.9 (±1.6)	14.3 (±1.5)	13.3 (±1.4)	0.004*
Preoperative Hb, mean (SD), g/dL	12.5 (±1.4)	12.9 (±1.5)	12.1 (±1.2)	0.011*
Postoperative Hb, mean (SD), g/dL	12.3 (±2.1)	12.4 (±2.0)	12.3 (±2.3)	0.786
Hb at Discharge, mean (SD), g/dL	10.9 (±1.7)	10.8 (±1.8)	10.9 (±1.5)	0.851
Change in Hb baseline to preoperative, mean (SD), g/dL	-1.3 (±0.9)	-1.2 (±0.9)	-1.3 (±0.8)	0.972
Change in Hb preoperative to postoperative, mean (SD), g/dL	-0.03 (±1.6)	- 0.58 (±1.3)	0.64 (±1.7)	0.002*

Female patients had lower pre-donation hemoglobin than male patients (13.16 + 1.12 vs. 14.71 + 1.48; p < 0.0001). However, amongst all the patients who were or were not transfused, there was no difference in the representation of either gender (p = 0.291).

Of the donated units, 90 (64%) were collected for surgeries performed by four surgeons. The most common surgeries for which PABD was requested were hepatic lobectomy for live donor liver transplant (47%), abdominal or laparoscopic myomectomy or laparoscopic hysterectomy for fibroids (8%), total hip or knee replacement or revision (8%), and partial nephrectomy or radical prostatectomy (8%).

Forty patients received transfusion of their autologous unit(s) peri-operatively. Of these transfused patients, 71% experienced an EBL of less than 500 mL. Post-operative Hb was greater than 10 g/dL for 80% (32/40) of the transfused PABD patients, and greater than 14 g/dL in six of these patients.

For liver transplant surgeries alone, transfusion of autologous units was predicted by lower pre-donation (p=0.001) and pre-surgery (p=0.010) Hb values. Among the other clinical specialties (general surgery, gynecology, hematology, kidney transplant, neurosurgery, otolaryngology, pediatric cardiothoracic surgery, thoracic surgery, urology, vascular surgery, cardiac surgery, orthopedic surgery) pre-donation and pre-surgery Hb values were not predictive of autologous transfusion and insufficient transfusion occurred to facilitate analysis of orthopedic, urology, and other surgical specialties.

Patients who received autologous transfusion had mean Hb values of 13.3 ± 1.4 g/dL prior to autologous donation and 12.1 ± 1.2 g/dL immediately prior to surgery. Patients who underwent PABD but were not transfused perioperatively had mean Hb values of 14.3 ± 1.5 g/dL prior to autologous donation and 12.9 ± 1.5 g/dL immediately prior to surgery (Table 1).

Of the total 141 donated units, 46 (33%) were transfused while 95 were discarded (67%). Table 2 illustrates the most common clinical services utilizing the PABD program, the number of PABD patients, units donated, and wastage rates. The wastage rate is defined as the percentage of autologous units donated but not transfused back to the donor, and therefore discarded.

**Table 2:** Preoperative Autologous Blood Donation Wastage by Clinical Service Abbreviation: PABD, preoperative autologous blood donation

Clinical Service	PABD units	Patients	Wastage Rate per Service
Liver Transplant	63 (45%)	61	37 (59%)
Gynecology	18 (13%)	11	16 (89%)
Orthopedic Surgery	14 (10%)	12	12 (86%)
Urology	12 (9%)	11	12 (100%)
Other	34 (23%)	23	18 (53%)

## Discussion

Significant inefficiencies in patient blood management were made apparent through examination of the PABD practice at our institution. PABD use was associated with high overall wastage of donated units and significant overtransfusion of participating patients. Wastage of autologous units remained high across all clinical specialties, suggesting a cause attributable to PABD rather than due to mismanagement of blood products within a specific clinical service. Similar reports of high wastage among autologous blood donation programs have been commonly reported across many studies (range 30%-54.4%) [5, 12-13], further supporting the presence of indelible inefficiencies within PABD practices.

Overtransfusion of autologous units was a common occurrence. Patients receiving autologous transfusions often experienced a low EBL and did not reach critically low Hb values (Hb < 7-8 g/dL) prior to transfusion. Furthermore, 80% of donors who later received transfusions reached a post-operative Hb > 10 g/dL. If restrictive transfusion practices aligning with AABB recommendations were applied to collected autologous units, wastage of collected units would predictably increase contributing further to inefficiencies in patient blood management [10].

The risks to patient safety of PABD are not limited to lack of benefit associated with overtransfusion. Donors of autologous units within our study presented with lower preoperative Hb than pre-donation Hb, a finding reported in many other studies [3-5]. Pre-operative anemia exposes patients to increased risk for postoperative morbidity and mortality among both cardiac and non-cardiac surgery patients [7-9]. Pre-operative anemia is also an independent risk factor for transfusions of any source [13]. Thus, it is possible participation in PABD may place patients at greater risk of experiencing adverse events related to pre-operative anemia and/or of needing blood transfusion during the peri-operative period.

## Conclusions

PABD practice was disproportionately represented in a minority of physician’s practices across multiple surgical specialties. The persistence of this practice highlights the current need for education of prescribing providers of superior alternatives for patient blood management and warrants evaluation of counseling patients are receiving regarding risks and benefits of PABD. While it can be argued the benefits of PABD may outweigh the harms for select patient populations (such as those with antibodies that complicate cross-matching of blood or those who object to allogeneic blood transfusion for strong personal reasons), these groups represent a small minority of the larger patient population.
